# Neuronal responses to face-like and facial stimuli in the monkey superior colliculus

**DOI:** 10.3389/fnbeh.2014.00085

**Published:** 2014-03-17

**Authors:** Minh Nui Nguyen, Jumpei Matsumoto, Etsuro Hori, Rafael Souto Maior, Carlos Tomaz, Anh H. Tran, Taketoshi Ono, Hisao Nishijo

**Affiliations:** ^1^System Emotional Science, Graduate School of Medicine and Pharmaceutical Sciences, University of ToyamaToyama, Japan; ^2^Primate Center and Laboratory of Neurosciences and Behavior, Department of Physiological Sciences, Institute of Biology, University of BrasíliaBrasilia, Brazil

**Keywords:** superior colliculus, subcortical pathway, face, face-like patterns, monkey

## Abstract

The superficial layers of the superior colliculus (sSC) appear to function as a subcortical visual pathway that bypasses the striate cortex for the rapid processing of coarse facial information. We investigated the responses of neurons in the monkey sSC during a delayed non-matching-to-sample (DNMS) task in which monkeys were required to discriminate among five categories of visual stimuli [photos of faces with different gaze directions, line drawings of faces, face-like patterns (three dark blobs on a bright oval), eye-like patterns, and simple geometric patterns]. Of the 605 sSC neurons recorded, 216 neurons responded to the visual stimuli. Among the stimuli, face-like patterns elicited responses with the shortest latencies. Low-pass filtering of the images did not influence the responses. However, scrambling of the images increased the responses in the late phase, and this was consistent with a feedback influence from upstream areas. A multidimensional scaling (MDS) analysis of the population data indicated that the sSC neurons could separately encode face-like patterns during the first 25-ms period after stimulus onset, and stimulus categorization developed in the next three 25-ms periods. The amount of stimulus information conveyed by the sSC neurons and the number of stimulus-differentiating neurons were consistently higher during the 2nd to 4th 25-ms periods than during the first 25-ms period. These results suggested that population activity of the sSC neurons preferentially filtered face-like patterns with short latencies to allow for the rapid processing of coarse facial information and developed categorization of the stimuli in later phases through feedback from upstream areas.

## Introduction

The superior colliculus (SC) is a multilayered structure in the mammalian midbrain. Its superficial layers (sSC) receive visual inputs from the retina (Leventhal et al., 1981; Perry and Cowey, 1984; Rodieck and Watanabe, 1993). The SC is thought to provide a subcortical pathway that is parallel to the extrastriate cortex and that bypasses the striate cortex (V1) in patients with V1 lesions (Weiskrantz, 1996; Berman and Wurtz, 2010, 2011; Pessoa and Adolphs, 2010; Kato et al., 2011). The sSC projects to the pulvinar and lateral geniculate nuclei, which have reciprocal connections with a number of cortical areas (Benevento and Fallon, 1975; Linke et al., 1999; Grieve et al., 2000; Kaas and Lyon, 2007; Schmid et al., 2010). Humans and monkeys with V1 lesions display residual visual functions in the blind area (i.e., blindsight) (Stoerig and Cowey, 1997; Yoshida et al., 2012). Human subjects with V1 lesions can respond differentially to the spatial localization of stationary and moving stimuli (Perenin and Jeannerod, 1975; Blythe et al., 1987), motion direction (Barbur et al., 1980; Perenin, 1991), line orientation (Weiskrantz, 1987), wavelength (Morland et al., 1999), and form (Perenin and Rossetti, 1996).

A number of neurophysiological studies have reported that monkey sSC neurons respond to a stationary light spot and its movements and that some neurons are motion direction sensitive (Goldberg and Wurtz, 1972). These neurons with specific receptive fields are topographically and retinotopically localized (Cynader and Berman, 1972; Dräger and Hubel, 1976). The response magnitude of sSC neurons has been shown to depend on the intensity and contrast of the visual stimuli with higher intensities and contrasts increasing responsiveness (Schneider and Kastner, 2005; Bell et al., 2006; Li and Basso, 2008). Furthermore, attention to stimuli increases visual responses (Wurtz and Mohler, 1976; Li and Basso, 2008). These characteristics of SC neurons may account for some residual functions that are observed after V1 lesions (i.e., spatial localization of stationary and moving stimuli, motion direction, etc.).

However, other evidence has indicated that this subcortical bypass route, including SC, might process stimuli forms, such as faces. Newborn babies with immature cortical systems preferentially orient toward faces with direct gazes and schematic face-like patterns (Johnson et al., 1991). Infant monkeys reared in isolation or without exposure to faces respond to pictures of conspecifics (Sackett, 1966; Sugita, 2008), and SC lesions in infant monkeys decrease their social behaviors among conspecifics (Maior et al., 2012). Convergent animal and human evidence suggests that newly hatched and dark-reared chicks, as well as human newborn babies, show preference for the same face-like patterns (three dark blobs on a bright oval) and human photo (Johnson and Horn, 1988; Rosa-Salva et al., 2010, 2011). These findings suggest the existence of innate face processing subcortical system that includes the SC and is common to many vertebrates, and also suggest that this system may not be sensitive to face differences among the species. Furthermore, non-invasive human studies of patients with blindsight have suggested that the subcortical route, including the SC, the pulvinar, and the amygdala, processes coarse [low spatial frequency (LSF)] images of faces (Morris et al., 2001; Vuilleumier et al., 2003). Although these studies suggest SC involvement in the processing of facial and face-like stimuli, most previous neurophysiological studies have examined moving dots, gratings, or simple patches. Consequently, evidence that sSC neurons process prototypical facial stimuli is lacking.

Since it has been reported that individual sSC neurons are not sensitive to shape (Schiller and Koerner, 1971; Goldberg and Wurtz, 1972), we hypothesized that population activity of sSC neurons could better discriminate these prototypical facial stimuli than activity of individual sSC neurons. It is reported that the sSC neurons had wide range of receptive fields from 2 to 20° with the deeper neurons having larger receptive fields (Goldberg and Wurtz, 1972; Li and Basso, 2008), and that sSC is retinotopically organized (Cynader and Berman, 1972). Therefore, when visual stimuli (5–7° × 5–7° in the present study) are presented in the central visual field, the stimuli would stimulate inside or a part of a receptive field of a given sSC neuron depending on eccentricity and size of the receptive field of the given neuron. Complex interaction between a stimulus and a receptive field could affect neuronal responses to the visual stimulus in a single neuronal level, and might result in different patterns of neuronal responses to visual stimuli in a population level. Since the subcortical visual pathway is implicated in some forms of facial information processing, the present hypothesis supposes that such activity patterns of all these sSC neurons based on such complex interaction could contribute to detection of prototypical facial stimuli. To test this hypothesis, we recorded sSC neuronal responses to these stimuli (human photos and face-like patterns) in monkeys, and picked up and analyzed the whole visual responses to determine whether population activity of these sSC neurons could contribute to detection of these prototypical stimuli (face-like patterns) to which human newborn babies and newly hatched chicks orient.

## Experimental procedures

### Subjects

Two adult (1 female and 1 male) macaque monkeys (*Macaca fuscata*), weighing 7.2–9.5 kg, were used in this experiment. Each monkey was individually housed with food available *ad-libitum*. The monkeys were deprived of water, and they received juice as a reward during the training and recording sessions. Supplemental water and vegetables were given after each day's session. In order to assess the monkeys' health, their weight was routinely monitored. The monkeys were treated in strict compliance with the United States Public Health Service Policy on Human Care and Use of Laboratory Animals, the National Institutes of Health Guide for the Care and Use of Laboratory Animals, and the Guidelines for the Care and Use of Laboratory Animals of the University of Toyama. Every effort was made to minimize the number of animals used and their suffering. The study had been approved by the Committee for Animal Experiments and Ethics at the University of Toyama.

In a shielded room, the monkey sat in a monkey chair that was located 68 cm away from the center of a 19-in computer display, which was used for the behavioral tasks during the training and recording sessions. A cathode ray tube (CRT) monitor was set so that its center was on the same horizontal plane as the monkey's eyes. The monkey chair was equipped with a response button, which was positioned so that the monkey could easily manipulate it. An infrared charge-coupled device camera, which was used for eye-movement monitoring, was firmly attached to the chair by a steel rod. During the training and recording sessions, the monkey's eye positions were monitored with a 33-ms time resolution by an eye-monitoring system (Matsuda, 1996). The juice reward was accessible to the monkey through a small spout that was controlled by an electromagnetic valve. A PsyScope system (Carnegie Mellon University, Pittsburgh, PA, USA) controlled the electromagnetic valve and sound signal, as well as the timing of the outputs to the CRT monitor.

### Visual stimuli

**Figure 1:**
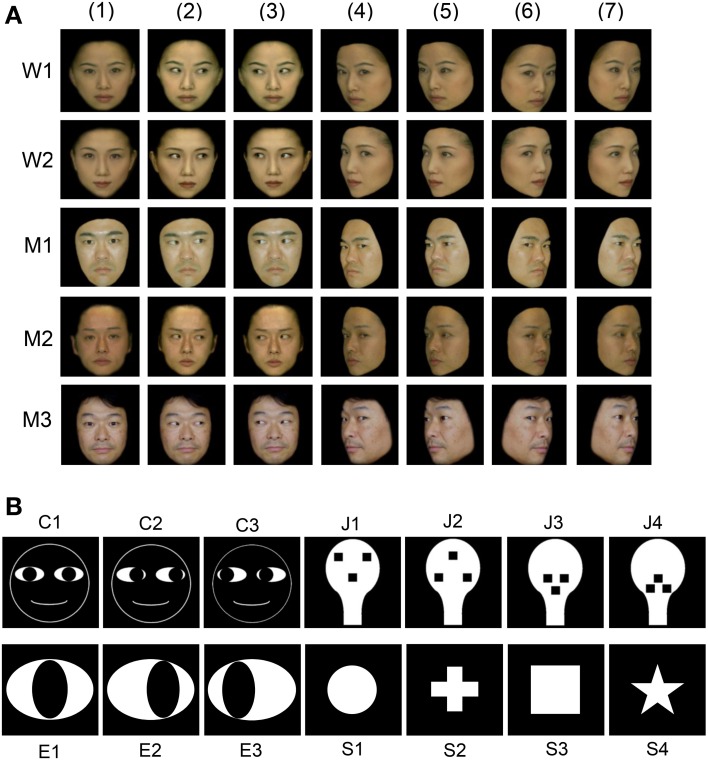
**Visual stimulus set used in the present study. (A)** Thirty-five facial photos of 5 different models, including 2 females (W1 and W2) and 3 males (M1, M2, and M3). The stimulus set for each model consisted of 7 faces with the following different head orientations and gaze directions: (1) frontal view with direct gaze; (2) frontal view with gaze to the right; (3) frontal view with gaze to the left; (4) left profile view with direct gaze; (5) left profile view with indirect gaze; (6) right profile view with direct gaze; and (7) right profile view with indirect gaze. **(B)** Fourteen artificial schematics, including 3 cartoon faces (C1–3) with three gaze directions, four face-like patterns (J1–4), three eye-like patterns with three gaze directions (E1–3), and four simple geometric patterns consisting of a circle, cross, square, and star (S1–4).

Figure 1A shows the stimulus set, which consisted of photos of human faces, that was used in the present study. These photos have been previously reported to activate monkey amygdala neurons (Tazumi et al., 2010). The facial photos, which were obtained with five human models, consisted of three head orientations: straight ahead (frontal face), 30° to the right (profile face), and 30° to the left (profile face). The frontal faces consisted of three gaze directions (directed toward and averted to the left or right of the monkey), and the profile faces comprised two gaze directions (directed toward and averted to the right and left of the monkey). The facial stimuli were 256 digitized color-scale images. The faces with averted gaze directions were artificially created from the faces with directed gazes by replacing the direct gazes in the eye region with averted gazes; thus, the only difference was the gaze direction. Stimuli were presented on a black background of 0.7 cd/m^2^ with their centers at the center of the display. The luminance of each stimulus was determined by measuring the luminance of the circular area (radius, 6.35 cm), including each stimulus inside the circle, by means of a luminance meter (BM-7A; Topcon Corporation, Tokyo, Japan) that was placed at 365 cm from the screen. The luminance of these stimuli ranged from 1.36 to 3.66 cd/m^2^, and the luminous intensity (total luminance) ranged from 16.4 to 44.2 mcd.

Figure 1B shows line drawings of the faces with three gaze directions (cartoon faces; C1–3), the eye-like patterns (E1–3), and the face-like patterns (J1–4) that newborn babies orient toward (Johnson et al., 1991). Since contrast of stimuli can modulate the responses of SC neurons (Li and Basso, 2008; Marino et al., 2012), we used 14 artificial figures with the same contrast. The luminance of the white and black areas inside these illustrations was 36.5 and 0.7 cd/m^2^, respectively, and the total luminance of the cartoon faces, eye-like patterns, and face-like patterns was 38.7, 188.6, and 179.3 mcd, respectively. In addition, for control stimuli, 4 simple geometric patterns (circle, cross, square, and star) were used. The luminance of the white areas inside the simple geometric patterns was 36.5 cd/m^2^, and the total luminance of the circle, cross, square, and star was 151.6, 96.0, 188.1, and 61.0 mcd, respectively. The cartoon faces, eye-like patterns, face-like patterns, and simple geometric patterns comprised digitized RGB images (8 bits per channel). These stimuli were displayed on a CRT monitor with a resolution of 640 × 480 pixels, and the size of the stimulus area was 5–7 × 5–7° that is comparable to receptive field of sSC neurons (Goldberg and Wurtz, 1972; Li and Basso, 2008). Some of the sSC neurons were further tested with scrambled and LSF-pass filtered images of the stimuli that elicited the strongest responses. Scrambled images were formed by cutting the original image into 286–441 pieces and randomly reassembling the fragments. LSF-pass filtered images were formed by low-pass filtering at 6 cycles per image.

### Behavioral tasks

**Figure 2:**
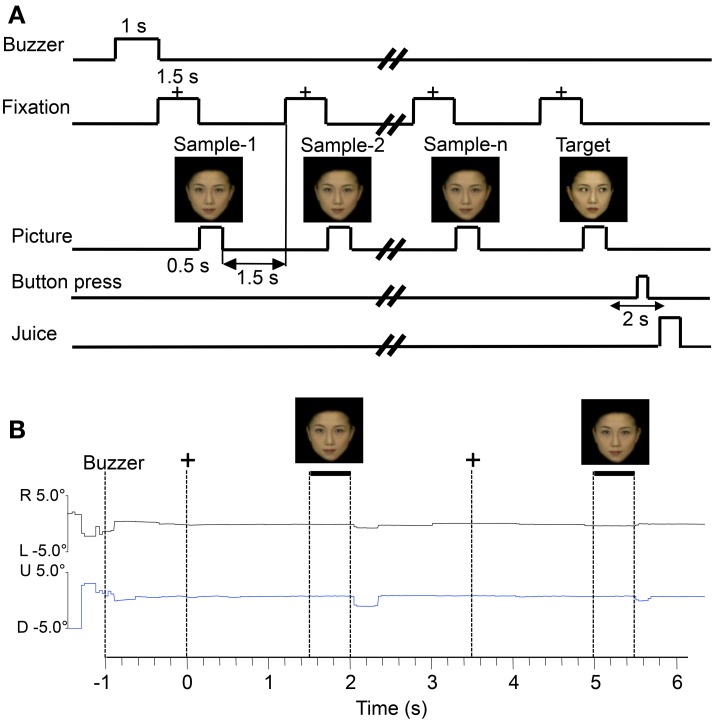
**Stimulus sequence in the delayed non-matching to sample (DNMS) task in which stimuli were sequentially presented with a delay between them (A) and examples of eye tracking data during the task (B).** +, fixation cross; R, rightward; L, leftward; U, upward; D, downward.

The monkeys were trained to perform a sequential delayed non-matching-to-sample task (DNMS) that required the discrimination of faces, face-like schematics, and simple patterns (Figures 1A,B). As illustrated in Figure 2A, the task was initiated by a buzzer tone. Then, a fixation cross appeared in the center of the display. When the monkeys fixated on the cross for 1.5 s within 0.5° window, a sample stimulus was presented for 500 ms (sample phase). The control phase was defined as the 100-ms period before the sample phase. When facial photos were used as sample stimuli, the gaze directions of the stimuli were either directed to or averted from the monkey. Then, after an interval of 1.5 s, the same stimulus appeared again for 500 ms between 1 and 4 times (selected randomly for each trial). Finally, a new stimulus with a different gaze direction was presented (target phase). When the target appeared, the monkey was required to press a button within 2 s in order to receive a juice reward (0.8 mL). The DNMS was used since the task forced the monkeys to be more engaged in the stimuli, which might generate greater visually evoked discharge rates. When the monkey failed to respond correctly during the target phase or to press the button before the target phase, the trials were aborted and a 620-Hz buzzer tone was presented. The intertrial intervals (ITI) were 15–25 s (Figure 2A). Figure 2B shows examples of eye tracking data during the DNMS task. Both subjects directed their eye gaze steadily on the fixation crosses and stimuli throughout the trials. Monitoring of eye movements and pupil radius was later used to rule out the trials in which the animals looked away or had their eyelids closed during stimulus presentation.

In the DNMS, the monkey compared a pair of stimuli in each trial (i.e., sample and target stimuli). Stimulus pairs consisted of the same category of stimuli, and, thus, only pairs of facial stimuli and pairs of geometric patterns were used (i.e., facial stimuli were not paired with geometric patterns). In the facial pairs, averted gazes were always paired with directed gazes, and stimulus pairs of gazes averted to the left and the right were not used. Furthermore, the facial stimuli that were presented in the target phase were the same as in the comparison phase, apart from gaze direction (i.e., same model and same head orientation). Thus, the monkeys were required to detect a difference in gaze direction (directed vs. averted gaze). For the geometric patterns (Figure 1B), only stimuli within the same category (cartoon faces, face-like patterns, eye-like patterns, and simple geometric patterns) were paired. Thus, a total of 72 stimulus pairs (for each of the five models: frontal faces, four pairs; profile faces, four pairs; cartoon faces, four pairs; face-like patterns, 12 pairs; eye-like patterns, four pairs; simple geometric patterns, 12 pairs) were used. These procedures facilitated the monkeys learning that a shift in gaze direction was an important clue for solving the task.

### Training and surgery

The monkeys were trained in the DNMS task for 3 h/day, 5 days/week. The monkeys required about 11 months of training in order to reach a 97% correct-response rate. After completion of this training period, a head-restraining device (a U-shaped plate made of epoxy resin) was attached to the skull under aseptic conditions (Nishijo et al., 1988a,b; Tazumi et al., 2010). The subject was anesthetized with a combination of medetomidine hydrochloride (0.5 mg/kg, i.m.) and ketamine hydrochloride (5 mg/kg, i.m.). The U-shaped plate was anchored with dental acrylic to titanium bolts that were inserted in the skull. We also implanted a reference pin, the location of which was based on the zero coordinates defined in the stereotaxic atlas of the brain of *Macaca fuscata* individuals (Kusama and Mabuchi, 1970). During the surgery, heart and respiratory functions and rectal temperature were monitored (LifeScope 14; Nihon Kohden Corporation, Tokyo, Japan). A blanket heater was used to keep body temperature at 36 ± 0.5°C. Antibiotics were administered topically and systemically for 1 week after the surgery in order to prevent infection. Two weeks after the surgery, the monkey was retrained while the head was painlessly fixed to the stereotaxic apparatus with the head-restraining device. The performance criterion (>85%) was again attained within 10 days.

### Stereotaxic localization of the SC for recording and histology

Before recording from the SC in each hemisphere, a marker that consisted of a tungsten wire (diameter: 500 μm) was inserted near the target area under anesthesia, and three-dimensional magnetic resonance imaging (3-D MRI) scans of the monkey head were performed. The 3-D pictures of the monkey brain with the marker were reconstructed by computer rendering. The 3-D stereotaxic coordinates of the target area were determined in reference to the marker in the 3-D reconstructed brain (Asahi et al., 2003, 2006). The superficial layers of the SC were determined from the surface (where the noises increased during insertion of the electrode) to 1 mm deeper vertically.

After the last recording session, several small marking lesions were created in the SC by passing 20–30 μA of anodal current for 30 s through an electrode that was placed stereotaxically. Subsequently, the monkeys were deeply anesthetized with an overdose of sodium pentobarbital (60 mg/kg, i.m.) and then perfused transcardially with 0.9% saline, which was followed by 10% buffered formalin. The brains were removed from the skulls and cut into 50-μm sections that contained the SC. The sections were stained with cresyl violet. The sites of the electrical lesions were determined microscopically. The location of each recording site was then calculated by comparing the stereotaxic coordinates of the recording sites with those of the lesions, and they were plotted on the actual tissue sections. The locations of visually responsive neurons in the two monkeys were compared on the basis of the shapes of the SC and replotted on the serial sections of the SC of 1 monkey, from 6.0 mm [anterior posterior (AP) 6.0] to 2.5 mm anterior (AP2.5) to the interaural line.

### Electrophysiological procedures and data acquisition

After the monkeys relearned the DNMS task at a rate greater than 85% correct, we commenced recording neuronal activity. Neuronal activity was recorded from each hemisphere in both subjects. A glass-insulated tungsten microelectrode (0.8–1.5 MΩ at 1 kHz) was stereotaxically inserted into the SC vertically to the orbitomeatal plane in a stepwise fashion by a pulse motor-driven manipulator (SM-21; Narishige Scientific Instrument Lab, Tokyo, Japan). Only neuronal activities with a signal-to-noise ratio (spike amplitude vs. noise level) greater than 3:1 were recorded. The analog signals of the neuronal activities were digitized at a 40-kHz sampling rate and stored in a computer through a multichannel acquisition processor (MAP; Plexon Inc., Dallas, TX, USA) system. The X-Y coordinates of eye position were digitized at a 1-kHz sampling rate and stored in the same system. The triggers for visual stimuli, juice rewards, and button pressing were stored through a digital input board of the same system. This information was also recorded on a data recorder (RT-145T; TEAC Corporation, Tokyo, Japan). The digitized neuronal activities were isolated into single units by their waveform components using the Offline Sorter program (Plexon Inc.). Superimposed waveforms of the isolated units were drawn in order to assess the variability throughout the recording sessions and then transferred to the NeuroExplorer program (Nex Technologies, Littleton, MA, USA) for further analysis. If the monkey exhibited signs of fatigue, such as closing the eyes for several seconds or moving the eyes or hands slowly, the experimental session was immediately terminated. In most cases, the unit recording experiment was terminated within 2–3 h.

### Analysis of the basic characteristics of SC neurons

**Figure 3:**
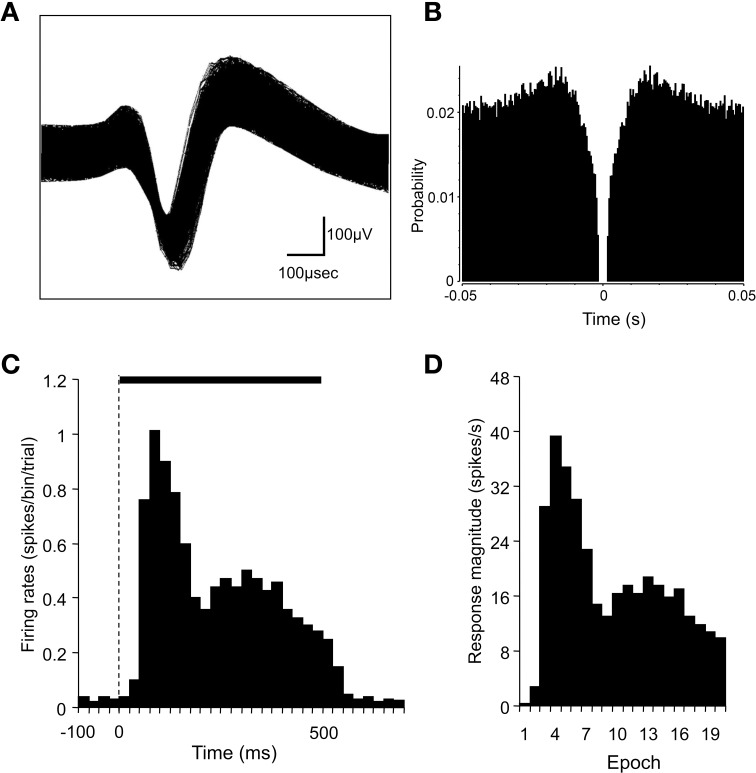
**Identification of superior colliculus (SC) neurons and the analysis of SC neuronal responses. (A)** An example of superimposed traces of a SC neuron. **(B)** Autocorrelograms of the neurons indicated in **(A)**. Bin width, 1 ms. The ordinate indicates probability where bin counts were divided by the number of spikes in the spike train. **(C)** A perievent histogram of the neuron indicated in **(A)**, showing responses to a visual stimulus. Bin width, 25 ms. The dashed line indicates the onset of stimulus presentation, and the horizontal bar indicates the duration of the stimulus (500 ms). **(D)** Magnitudes of the SC neuronal responses indicated in **(C)**. The stimulus duration was divided into 20 epochs (25 ms each). The response magnitudes (spikes/s) were defined as follows: the mean firing rate in each epoch minus the mean firing rate during the 100-ms period before stimulus onset.

Spike sorting was performed with the off-line sorter program for cluster analysis (Off-line sorter, Plexon Inc.). Each cluster was checked manually in order to ensure that the cluster boundaries were well separated and that the waveform shapes were consistent with the action potentials. For each isolated cluster, an autocorrelogram was constructed, and only units with refractory periods greater than 1.2 ms were used for further analyses. Finally, superimposed waveforms of the isolated units were drawn in order to check the consistency of the waveforms. Figures 3A,B show examples of the superimposed waveforms of a SC neuron and its autocorrelogram, respectively. This autocorrelogram indicated that the refractory period of the neuron was 2–3 ms throughout the recording sessions, which suggested that these spikes were recorded from a single neuron.

We analyzed single neuronal activity during the following two periods: 100 ms before (*pre*) and 500 ms after (*post*) the onset of stimulus presentation in the sample phase. The baseline firing rate was defined as the mean firing rate during the 100-ms *pre* period. Significant excitatory or inhibitory responses to each stimulus were defined by a Wilcoxon signed-rank (WSR) test (*p* < 0.05 for statistical significance) of the neuronal activity between the 100-ms *pre* and the 500-ms *post* periods. Furthermore, in order to investigate the temporal changes in the neuronal responses, the 500-ms *post* period was divided into twenty 25-ms epochs. The mean neuronal firing rate was calculated for each of these epochs. The response magnitude was defined as follows: the mean firing rate in each epoch minus the mean firing rate during the 100-ms *pre* period. Figures 3C,D show a perievent-summed histogram of responses from the same neuron that is shown in Figures 3A,B to a facial photo **(C)** and the response magnitudes in the 20 epochs converted from this histogram **(D)**.

For each neuron, the response magnitudes during the visual stimulation period (for the whole 500-ms period and for each epoch) for all visual stimuli were analyzed by One-Way ANOVA (one factor with 49 levels) (*p* < 0.05). Neurons with a significant main effect were defined as differential neurons. For each facial model, One-Way ANOVA was also performed. Responses to three frontal faces with three gaze directions and those to right and left profile faces with two gaze directions were compared by Tukey *post-hoc* tests (*p* < 0.05). Neurons with significantly different responses toward gaze directions were defined as gaze-differential neurons (Tukey tests, *p* < 0.05). Head orientation-differential neurons were defined as those in which *post-hoc* tests (Tukey tests, *p* < 0.05) indicated that there were significant differences in the responses among the three head orientations (three frontal faces, two left profiles, and two right profiles) regardless of gaze directions. For other stimulus categories (cartoon faces, eye-like patterns, face-like patterns, and simple geometric patterns), One-Way ANOVAs were also performed within the same stimulus category. Neurons with significant a main effect were defined as cartoon face-differential, eye-like pattern-differential, face-like pattern-differential, and simple geometric pattern-differential neurons, respectively.

Stimulus information that was conveyed by visually responsive neurons (bits/s) was computed as described in previous studies (Skaggs et al., 1993; Panzeri et al., 1996). These parameters were calculated as follows:

$$\text{I }=\sum_{i\;=\;1}^{n}\lambda \text{i p}\text{​}\left(\text{i}\right)\;{\text{log}}_{2}\frac{\lambda \text{i}}{\lambda }$$

Where I is the information rate of the neuron in bits per second, i is the stimulus number, λi is the response (mean firing rate) to the stimulus i, λ is the total average response, and p(i) is the probability of the stimulus. The neuronal activity of visually responsive neurons conveyed stimulus information, and consequently, visually responsive neurons displayed high values for this parameter. Furthermore, information for the specific stimulus sets (face-like patterns and visual stimuli other than face-like patterns) was also computed. These parameters were compared across different epochs by repeated measures One-Way ANOVA at a significance level of *p* < 0.05. *Post-hoc* comparisons were performed using the Bonferroni method with a significance level of *p* < 0.05.

In addition, we analyzed the response latency to each visual stimulus. For each neuron, 1 perievent histogram was constructed with the entire set of data for all trials and all stimuli. Neuronal response latency was defined as the interval from the onset of stimulus presentation to the time at which the neuronal firing rate exceeded the mean ± 2 SD of the baseline firing rate. Furthermore, for each neuron, individual perievent histograms were constructed with data for each of the different stimulus categories. We compared the latencies to various stimulus categories in order to determine whether the characteristics of the specific visual stimuli could modulate the latencies of the SC neurons. All neuronal response latencies were compensated for the delay between the trigger signals of the visual stimuli and the signals taken from a photodiode placed on the face of the video display. All data were expressed as mean ± s.e.m.

### Multivariate analysis of visual responses of the sSC neurons

Multidimensional scaling (MDS) is a method that is used to simplify the analysis of relationships that exist within a complex array of data. MDS constructs a geometric representation of the data in order to show the degree of the relationship between stimuli that are represented by the data matrix (see Young, 1987 for more details). MDS has been used to examine taste relationships in the gustatory system (Nishijo and Norgren, 1990, 1991), face categorization in the inferotemporal cortex (Young and Yamane, 1992), and spatial discrimination in the septal nuclei (Nishijo et al., 1997) with data matrices representing neural activity in response to the particular stimulus array (i.e., taste solutions, photos of faces, and photos of locations, respectively). In the present study, the 49 visual stimuli were used to elicit neural activity in sSC neurons.

Data matrices of neural activity in a 112 × 49 array derived from the 112 visually responsive neurons were generated. Euclidean distances as dissimilarity between all possible pairs of two visual stimuli were calculated by using the visual responses of the 112 sSC neurons. Then, the MDS program (PROXSCAL procedure, SPSS statistical package, version 16) positioned the visual stimuli in the 2-dimensional space with the distances between the stimuli representing the original relationships (i.e., Euclidean distances in the present study) (Shepard, 1962; Kruskal, 1964).

## Results

### Responses to the visual stimuli

**Figure 4:**
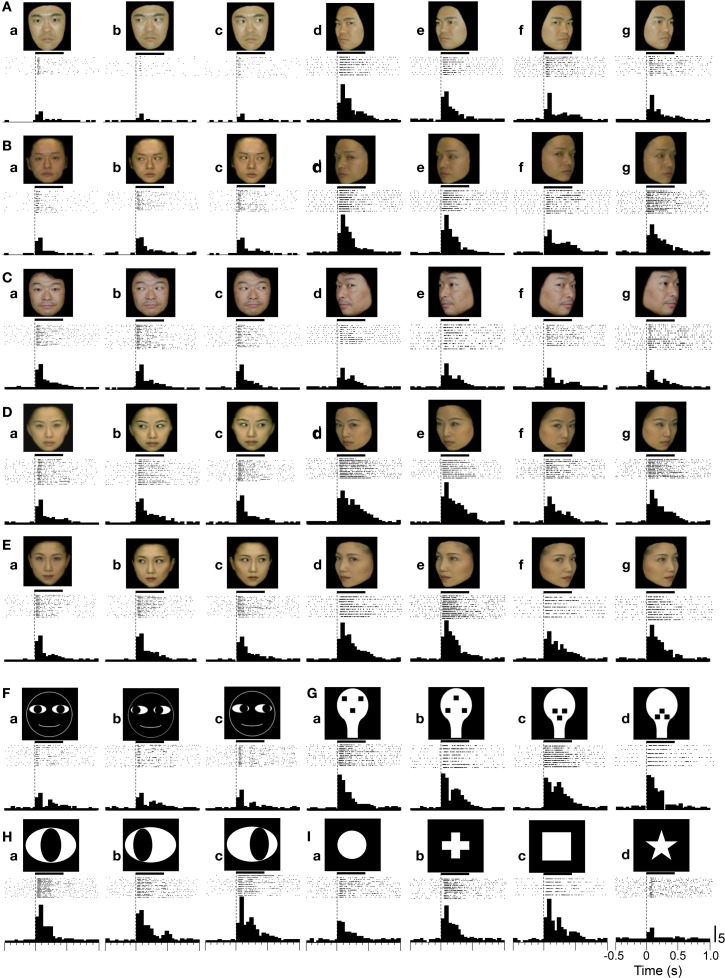
**An example of a superficial SC (sSC) neuron that responded to the visual stimuli. (A–E)** Responses to the facial photos. **(F–I)** Responses to the face cartoons **(F)**, face-like patterns **(G)**, eye-like patterns **(H)**, and simple geometric patterns **(I)**. Horizontal bars above the raster displays indicate the stimulus presentation period (500 ms). The vertical dotted line in each of the raster displays and histograms indicate the stimulus-onset point. Calibration at the right bottom of the figure: number of spikes per trial in each bin. Bin width, 50 ms.

Recordings were made from a total of 646 neurons from the SCs of two monkeys. Of these, the 41 neurons that were recorded from the intermediate layers of the SC were excluded from the analysis, and 605 neurons from the sSC were analyzed. Of the 605 neurons, 216 responded to the visual stimuli. Of these 216, 112 neurons were tested with all of the visual stimuli. Figure 4 shows an example of a sSC neuron that responded to various visual stimuli. The activity of the neuron increased sharply in response to the onset of the stimuli, then decreased rapidly, and then gradually increased again. This pattern of changes in neuronal activity formed the following two response phases: an early rapid response phase and a late gradual response phase. This neuron responded differentially to the head orientation of the human photos; the left profiles elicited the strongest responses (except for model C) while this neuron was less sensitive to frontal faces. Of the non-facial stimuli, this neuron showed comparable responses to face-like patterns (Figures 4Ga–d), eye-like patterns (Figures 4Ha–c), and square figures (Figure 4Ic).

**Figure 5:**
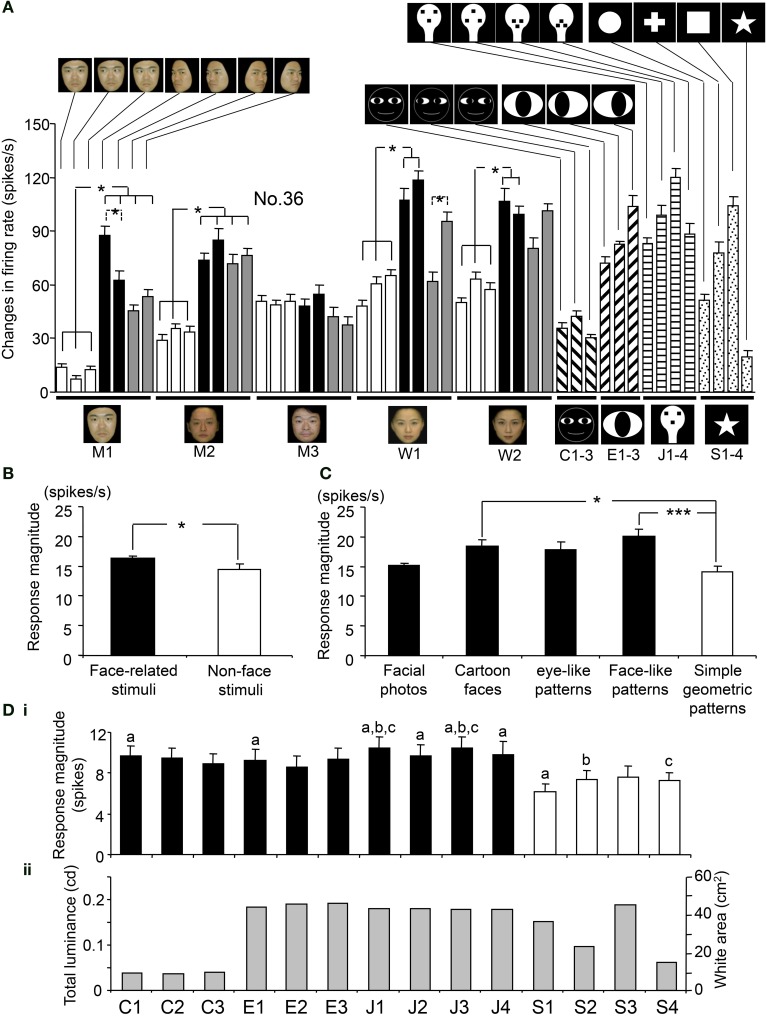
**Response magnitudes during stimulus presentation (500 ms) to the visual stimuli. (A)** Comparison of the response magnitudes to all of the visual stimuli during stimulus presentation (500 ms) for the neuron shown in Figure 4. All of the 49 stimuli elicited significant excitatory responses [Wilcoxon signed-rank (WSR), *p* < 0.05]. For each facial model, the response magnitudes to the profile faces were significantly greater than those to frontal faces (except model M3). The response magnitudes to the face-like patterns (J1-4) and the eye-like patterns (E1-3) were significantly greater than those to the cartoon faces (C1-3) (Tukey tests after One-Way ANOVA, *p* < 0.05). For each model, the responses to the stimuli with different head orientations and gaze directions are similarly aligned from the left, as for model M1. **(B)** Comparison of response magnitudes of the 112 visually responsive neurons to the face-related stimuli (facial photos, cartoon faces, eye-like patterns, and face-like patterns) and non-face stimuli (simple geometric patterns). The mean response magnitude to the face-related stimuli was larger than that to the non-face patterns [*F*_(1, 5486)_ = 3.96, *p* < 0.05]. **(C)** Comparison of response magnitudes of the 112 visually responsive neurons to the five stimulus categories. The face-like patterns and cartoon faces elicited stronger responses than the simple geometric patterns (Tukey tests, *p* < 0.001 and 0.05, respectively after One-Way ANOVA). **(D)** Relationships between response magnitudes **(i)** and total luminances (stimulus sizes) **(ii)** of the white and black stimuli. (a–c) significant difference between the stimuli indicated by the same letters (Bonferroni tests after repeated measures One-Way ANOVA, *p* < 0.05). Furthermore, there was no significant correlation between the total luminance (stimulus size) and response magnitude (Pearson's correlation coefficient, *r* = 0.148, *p* > 0.05). ^*^p < 0.05; ^***^p < 0.001 (Tukey tests after One-Way ANOVA).

Figure 5A shows the response magnitudes of the neuron shown in Figure 4 during stimulus presentation (500 ms) of all of the visual stimuli. There were significant differences in the response magnitudes to the various visual stimuli [*F*_(48, 591)_ = 16.17, *p* < 0.001]. Subsequent *post-hoc* tests indicated that the response magnitudes to the left-averted faces were significantly greater than those to the frontal faces (Bonferroni test, *p* < 0.05), except for model C. This neuron also showed differential responses to the gaze direction of models A and D (Bonferroni test, *p* < 0.05). The response magnitudes to the eye-like patterns **(Ha–c)** and the face-like patterns **(Ga–d)** were significantly greater than those to the cartoon faces **(Fa–c)** (Tukey test, *p* < 0.05). Of the 112 sSC neurons tested, 111 displayed differential responses to the various stimuli (One-Way ANOVA, *p* < 0.05).

### Population responses to the visual stimuli in the sSC

The overall mean responses indicated that the sSC neurons responded stronger to the face-related stimuli (facial photos, cartoon faces, eye-like patterns, and face-like patterns) than the non-face stimuli (simple geometric patterns). Figure 5B illustrates of the mean response magnitudes of the 112 visually responsive neurons during stimulus presentation (500 ms) to the face-related and non-face stimuli. The mean response magnitude of the 112 visually responsive neurons to the face-related stimuli was significantly larger than that to the non-face stimuli [*F*_(1, 5486)_ = 3.96, *p* < 0.05]. Figure 5C shows the mean response magnitudes of the 112 visually responsive neurons to the five stimulus categories. There were significant differences in response magnitudes to the five stimulus categories [*F*_(4, 5483)_ = 9.92, *p* < 0.001]. The cartoon faces and face-like patterns elicited stronger responses than the simple geometric patterns (Tukey tests, *p* < 0.05 and 0.001, respectively). These results indicate that the sSC neurons responded well to face-related stimuli.

However, previous studies reported that, unlike neurons in the geniculocortical visual pathway, population activity of sSC neurons respond best to smaller stimuli (2° or smaller in hamsters) or stimuli with optimal size (6–8° in mice) when different sizes of light spots were used as visual stimuli (Schiller and Stryker, 1972; Razak and Pallas, 2006; Wang et al., 2010). Therefore, the above present results could be ascribed to the differences in stimulus sizes. Figure 5D indicates relationships between response magnitudes and stimulus sizes of the white and black stimuli. In these white and black stimuli, stimulus sizes (sizes of the white areas) are correlated with total luminance (*p* = 0.001; *r* = 0.98). Among the simple geometric patterns, area of S4 is the smallest, which is comparable to the cartoon faces, while area of S3 is the largest, which is comparable to the face-like patterns. There were significant differences in response magnitudes among the 14 stimuli [*F*_(13, 1554)_ = 2.533, *p* < 0.05; repeated measures One-Way ANOVA]. The simple geometric patterns (S1, S2, and S4) elicited smaller responses than some of the cartoon faces, eye-like patterns and face-like patterns (Bonferroni tests, *p* < 0.05). S3 also tended to elicited smaller responses than some of the face-like patterns (J1 and J3) (Bonferroni tests, *p* < 0.1). This indicated that the simple geometric patterns elicited smaller responses regardless of their stimulus sizes, and that there was no optimal stimulus size at least among these 14 stimuli. Furthermore, there was no significant correlation between the stimulus size and response magnitude (Pearson's correlation coefficient, *r* = 0.148, *p* > 0.05). These results indicate that differential responses of the sSC neurons were not ascribed to stimulus size at least in population data.

### Response patterns of individual sSC neurons

Of these 112 visually responsive neurons, 28 neurons responded differentially to the gaze directions in the frontal or profile faces of at least one of the facial models (gaze-differential), and 41 responded differentially to head orientation (head orientation-differential). Differential responses to the gaze direction of the cartoon faces were displayed by 29 neurons (cartoon face-differential) and to the gaze direction of the eye-like patterns by 32 neurons (eye-like pattern-differential). Forty-eight and 46 neurons responded differentially to the face-like patterns (J1–4) (face-like pattern-differential) and to the simple geometric patterns (simple geometric pattern-differential), respectively. A previous study demonstrated that the mean response magnitudes in the amygdala toward the facial photos with direct gazes were significantly larger than those to facial photos with averted gazes (Tazumi et al., 2010). We analyzed the sSC responses in the same manner. However, the difference in the response magnitudes to these two different gaze directions was not statistically significant in the sSC (paired *t*-test, *p* > 0.05).

### Responses to the scrambled and LSF-filtered stimuli

**Figure 6:**
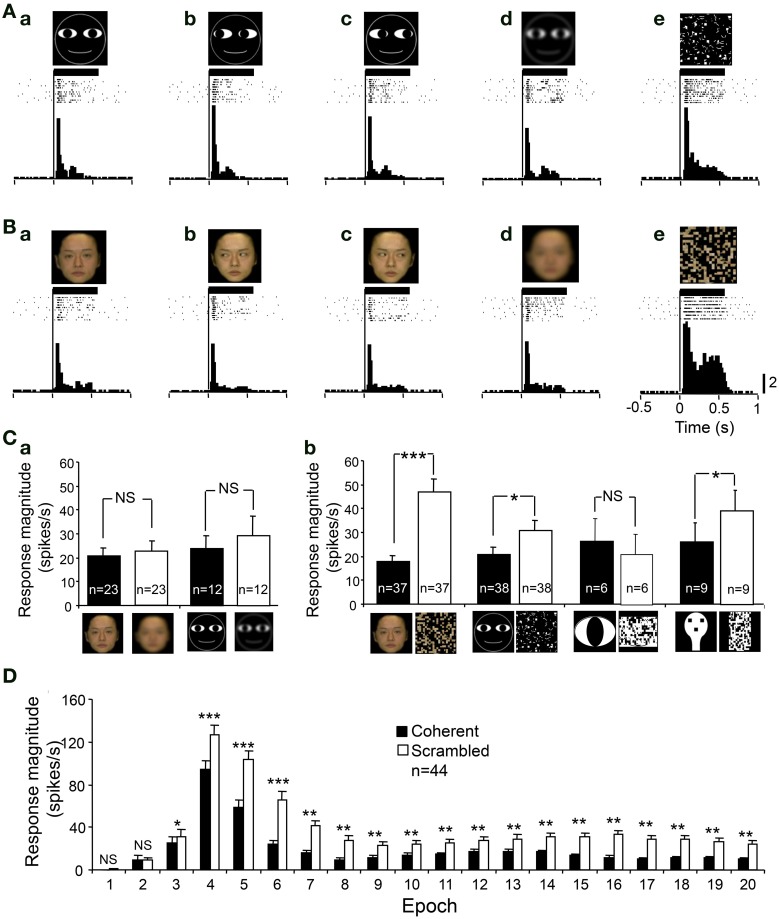
**An example of a sSC neuron tested with the scrambled and low spatial images. (A)** The neuron responded to the cartoon faces **(Aa–c)**. The neuron responded similarly to the low spatial frequency (LSF)-pass filtered image **(Ad)**, but more strongly to the scrambled image **(Ae)**. **(B)** The neuron responded to the human frontal faces **(Ba–c)**. The neuron responded similarly to the LSF-pass filtered image **(Bd)**, but more strongly to the scrambled image **(Be)**. Calibration at the right bottom of the figure: number of spikes per trial in each bin. Bin width, 25 ms. **(C)** Effects of LSF-filtering **(a)** and scrambling **(b)** of the photographic facial stimuli and non-photographic facial stimuli. ^***^p < 0.001; ^*^p < 0.05; NS, *p* > 0.05. **(D)** Effects scrambling of the stimuli across 20 epochs. Scrambling significantly enhanced the responses from epoch 3 to epoch 20 (paired *t*-test, ^***^p < 0.001; ^**^p < 0.01; ^*^p < 0.05; NS, *p* > 0.05). Other descriptions are the same as for Figure 4.

In order to analyze whether the visual responses were dependent on a coherent pattern and/or spatial frequency of the visual stimuli, we compared the responses to intact optimal stimuli with those to the scrambled and LSF images of the same stimuli. Figures 6A,B shows an example of a sSC neuron that was tested with scrambled and LSF images. The responses of this neuron were enhanced by scrambled images, but not by LSF images. Statistical analyses indicated that LSF-pass filtering did not significantly affect the responses to the facial photos (paired *t*-test, *p* > 0.05) and to the non-photographic stimuli (paired *t*-test, *p* > 0.05) (Figure 6Ca). However, scrambling significantly increased the responses to the facial photos (paired *t*-test, *p* < 0.001), cartoon faces (paired *t*-test, *p* < 0.05), and face-like patterns (paired *t*-test, *p* < 0.05) except the eye-like stimuli (paired *t*-test, *p* > 0.05) (Figure 6Cb). Figure 6D shows the effects of scrambling across 20 epochs, and significant enhancement was observed from epochs 3 to 20 (paired *t*-test; ^***^p < 0.001; ^**^p < 0.01; ^*^p < 0.05). These results indicated that the effects of scrambling were evident in later, but not in early, epochs.

### Response latencies of sSC neurons

**Figure 7:**
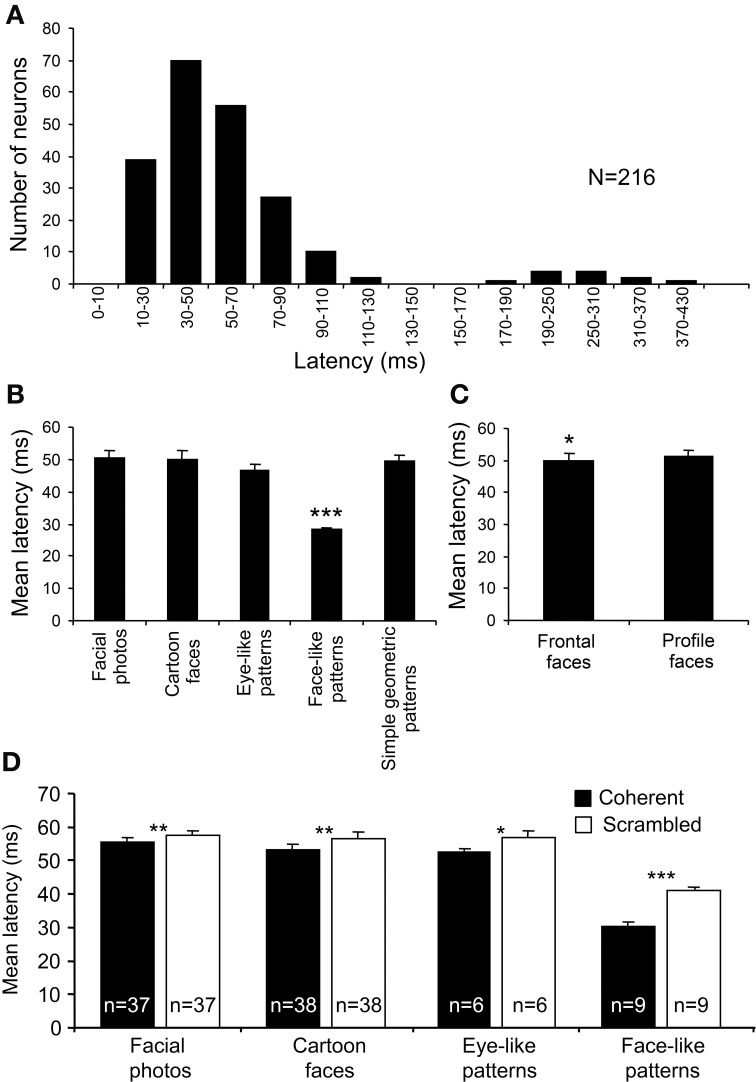
**Response latencies of the 216 visually responsive sSC neurons. (A)** The distribution of the mean response latencies. The distribution of the latencies formed two peaks: a short-latency group (10–130 ms) and a long-latency group (170–430 ms). **(B)** Comparison of the response latencies among the stimulus categories in the short-latency group. The mean response latencies to the face-like patterns (J1–4) were significantly shorter than those to the other stimulus categories (^***^p < 0.001; Bonferroni tests after repeated measures One-Way ANOVA). **(C)** Comparison of the response latencies between the head orientations. The mean response latencies to the frontal faces were significantly shorter than those to the profile faces (^*^p < 0.05; paired *t*-test). **(D)** Effects of scrambling of the images on response latencies. Scrambling significantly increased the mean response latencies (^*^p < 0.05; ^**^p < 0.01; ^***^p < 0.001; paired *t*-test).

Response latencies were analyzed using all 216 of the visually responsive neurons. Figure 7A shows the mean response latencies of the sSC neurons to various visual stimuli. The distribution of the latencies formed the following two peaks: a short-latency group (10–130 ms) and a long-latency group (170–430 ms). The mean latency of the short-latency group was 52.95 ± 1.71 ms (mean ± s.e.m.). Because the long-latency group elicited very weak responses (data not shown), the long latencies in this group might be ascribed to inadequate stimulation, and, thus, the present visual stimuli might have not been optimal for this group.

In order to investigate how the configuration of the visual stimuli modulated the response latencies, we analyzed the response latencies to each category of visual stimuli (Figure 7B). In the short-latency group, there were significant differences in the response latencies to the various stimulus categories [One-Way ANOVA; *F*_(4, 380)_ = 19.33, *p* < 0.001]. Multiple *post-hoc* comparisons indicated that the mean response latencies to the face-like patterns (J1–4) were very short (28.13 ± 0.90 ms) and shorter than those to the other categories (Tukey test for *post-hoc* comparison, *p* < 0.001). The differences in response latencies among the five stimulus categories could be ascribed to differences in total luminance. Nonetheless, the total luminance of the face-like patterns was smaller than those for square and eye-like stimuli, and the luminance of the white areas of the face-like patterns was the same as that of the simple geometric patterns. These findings indicate that specific early responses to the face-like patterns were not due to differences in total luminance or luminance. Furthermore, there was a significant difference in the response latencies to the different head orientations as well (Figure 7C). The mean response latency to the frontal faces (49.96 ± 2.09 ms) was significantly shorter than that to the profile faces (51.17 ± 2.05 ms) (paired *t*-test, *p* < 0.05).

In addition, scrambling of the images consistently increased the response latencies to facial photos (paired *t*-test, *p* < 0.01; *n* = 37), cartoon faces (paired *t*-test, *p* < 0.01; *n* = 38), eye-like patterns (paired *t*-test, *p* < 0.05; *n* = 6), and face-like patterns (paired *t*-test, *p* < 0.001; *n* = 9) (Figure 7D). These findings also indicate that short response latencies to the face-like patterns were dependent on coherent images. However, a previous study reported that, when different points within the receptive fields were stimulated by a light spot, response latencies were dependent on the locations within the receptive fields in some SC neurons, while responses latencies were constant all over the receptive fields in other SC neurons (Harutiunian-Kozak et al., 1973). Nevertheless, the latency results of scrambling might preclude the possibility that the face-like patterns stimulated the best locations within the receptive fields, since both the original and scrambled images stimulated almost the same visual fields, while the response latencies to the scrambled images were longer than those to the original images.

### Temporal changes in the response magnitudes to the visual stimuli

**Figure 8:**
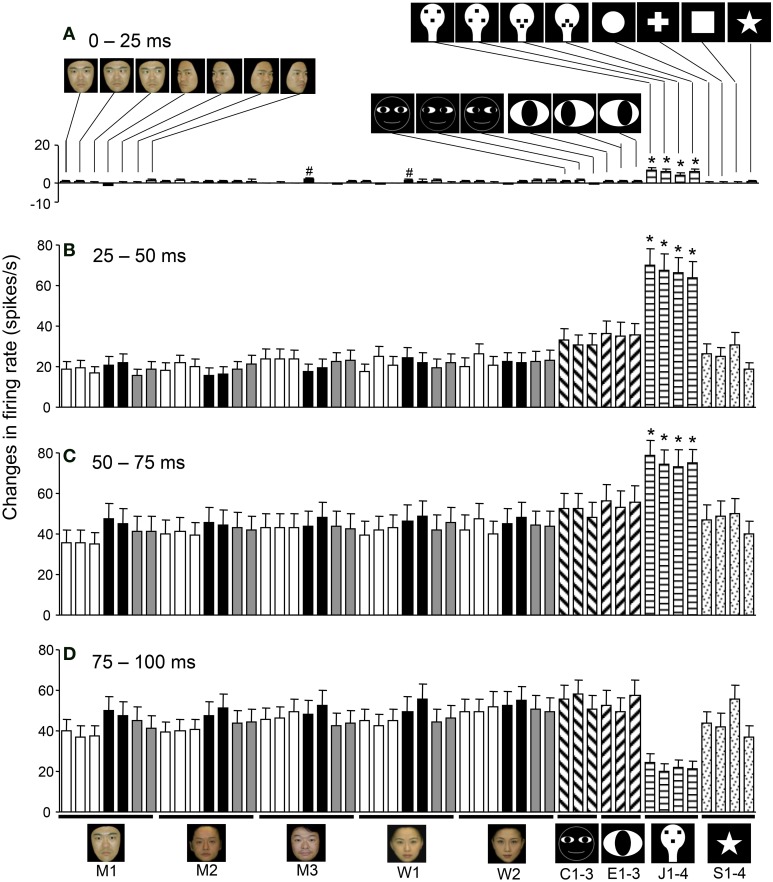
**Temporal changes in the mean response patterns of the 112 visually responsive neurons across the different epochs. (A)** Comparison of the response magnitudes to the 49 visual stimuli in epoch 1. In epoch 1, the face-like patterns (J1–4) elicited stronger responses than the other visual stimuli, except for the 2 stimuli indicated by # (Tukey tests after One-Way ANOVA, *p* < 0.05). **(B)** Comparison of the response magnitudes toward the 49 visual stimuli in epoch 2. In epoch 2, the response magnitudes to all visual stimuli increased compared to epoch 1 (paired *t*-test, *p* < 0.001). The response magnitudes to the face-like patterns were stronger than those to all of the other stimuli (Tukey tests after One-Way ANOVA, *p* < 0.05). **(C)** Comparison of the response magnitudes to the 49 visual stimuli in epoch 3. The response magnitudes to the face-like patterns were stronger than those to all of the other stimuli (Tukey tests after One-Way ANOVA, *p* < 0.05). **(D)** Comparison of the response magnitudes for the 49 visual stimuli in epoch 4. ^*^p < 0.05. Other descriptions are the same as those for Figure 5.

Figure 8 shows the mean response magnitudes of the 112 visually responsive neurons in the first 4 epochs. In epoch 1, the face-like patterns elicited stronger responses than the other visual stimuli (except for the two stimuli indicated with a #) (Tukey tests after One-Way ANOVA, *p* < 0.05). In epochs 2 and 3, the response magnitudes to all visual stimuli increased; the mean response magnitude to each stimulus was significantly larger than in epoch 1 (paired *t*-test, *p* < 0.01), and the face-like patterns continued to elicit stronger responses than the other visual stimuli did (Tukey tests after One-Way ANOVA, *p* < 0.05). These results indicated that sSC neurons were more sensitive to the visual stimuli from epoch 2. These changes in responsiveness across the various visual stimuli were not uniform at the single neuron level; the individual neurons displayed differential responses to various stimuli depending on the neurons.

### Multivariate analysis of sSC neuronal responses

**Figure 9:**
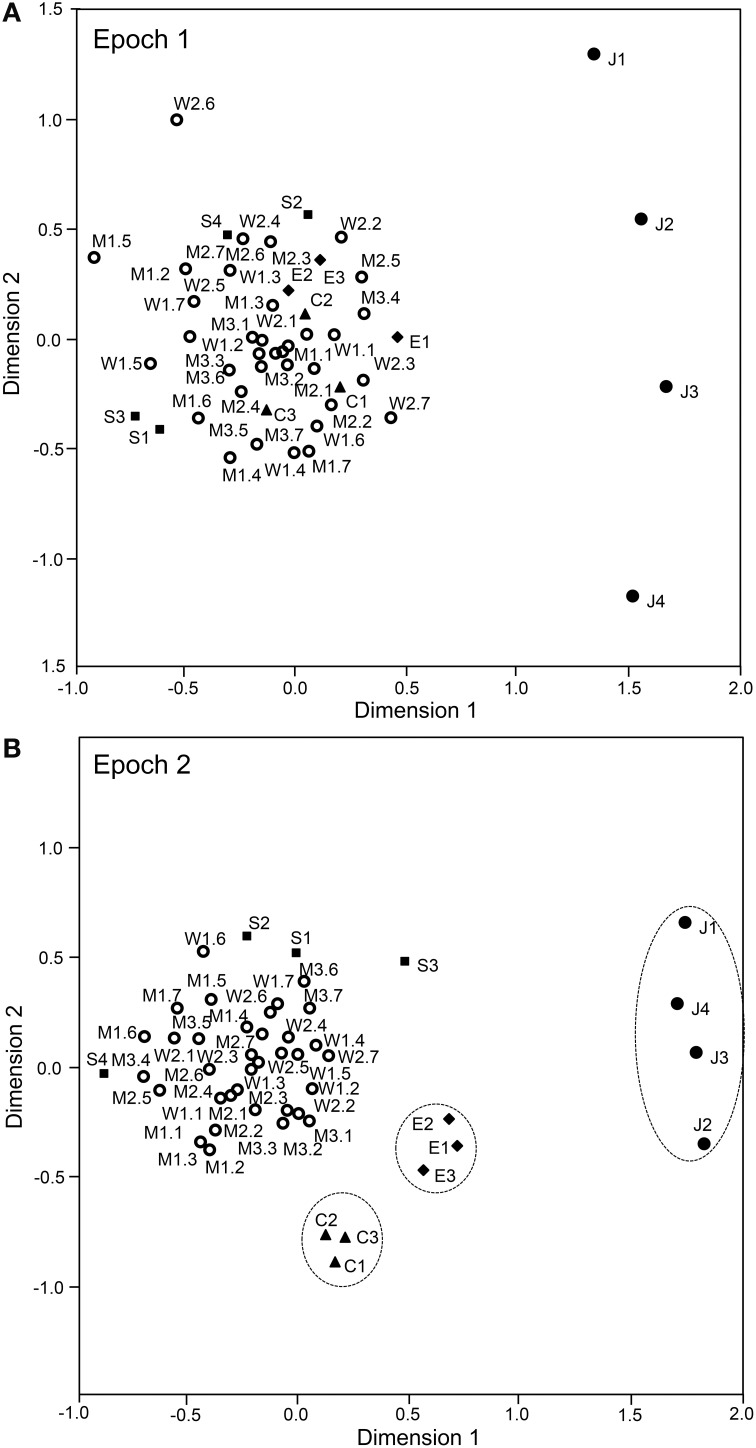
**Distributions of the 49 visual stimuli in the two-dimensional space resulting from multidimensional scaling in epoch 1 (A) and epoch 2 (B).** In epoch 1 **(A)**, 1 cluster that includes stimuli other than the face-like patterns (J1–4) was recognized. The face-like-patterns were located separately from this big cluster. In epoch 2 **(B)**, the following four groups were recognized: 1 large group that consisted of various stimuli, including the facial photos and simple geometric patterns, and three small groups corresponding to the face-like patterns, cartoon faces, and eye-like patterns. C1–3, three cartoon faces; J1–4, four face-like patterns; E1–3, three eye-like patterns; S1–4, simple geometric patterns; W1–2, female models; M1–3, male models. The numbers from 1 to 7 after the model numbers and colons indicate the head orientations and the gaze directions indicated in Figure 1A.

**Figure 10:**
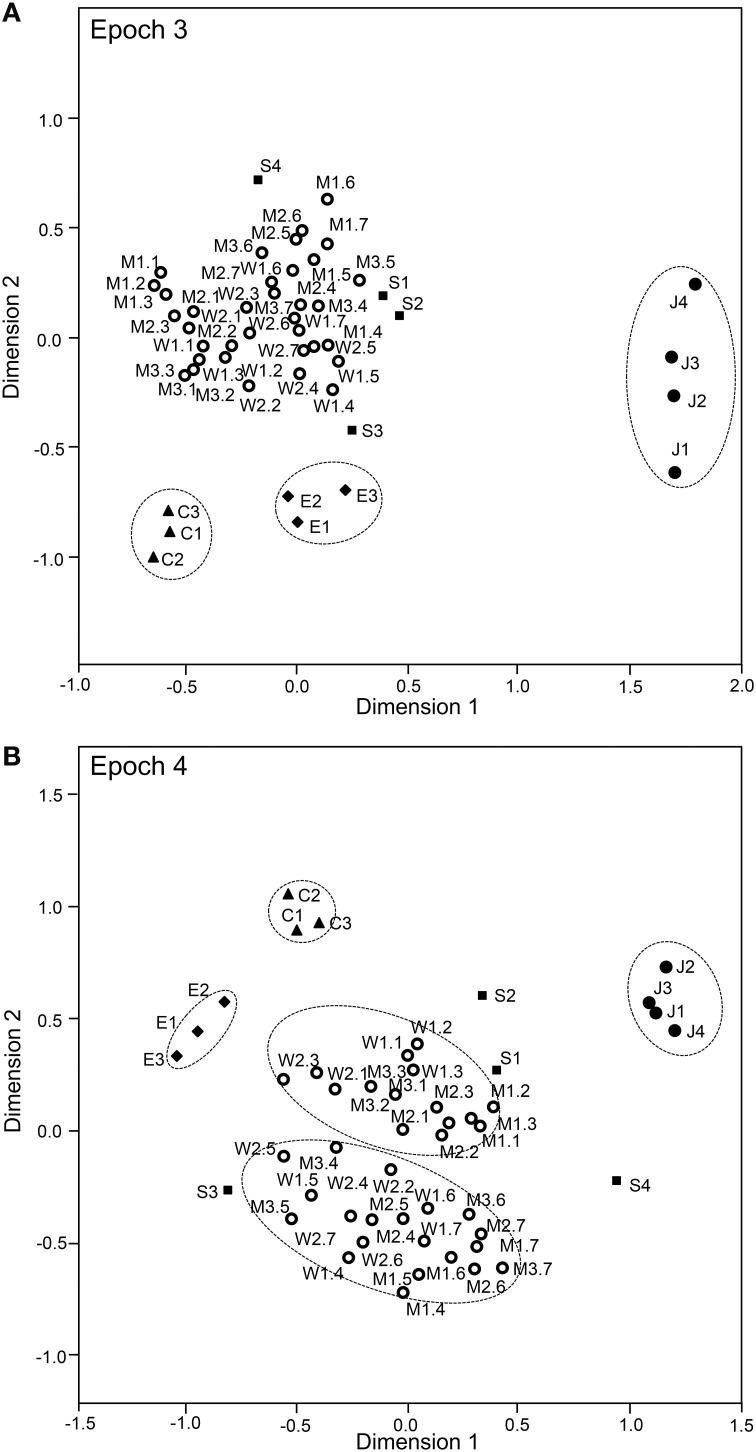
**Distributions of the 49 visual stimuli in the two-dimensional space resulting from multidimensional scaling in epoch 3 (A) and epoch 4 (B).** In epoch 3 **(A)**, four groups were recognized: one large group that consisted of various stimuli, including the facial photos and simple geometric patterns, and three small groups corresponding to the face-like patterns, cartoon faces, and eye-like patterns. In epoch 4 **(B)**, five groups were recognized: two large groups of neurons responding to stimuli corresponding to the frontal faces and profiles and three small groups of neurons responding to stimuli corresponding to the face-like patterns, cartoon faces, and eye-like patterns. Other descriptions are the same as those for Figure 11.

The above analyses suggested that sSC neurons specifically encode face-like patterns in epoch 1 and supplementary information in later epochs. The data sets of the response magnitudes recorded from the 112 sSC neurons in the first 4 epochs were subjected to a MDS analysis (Figures 9, 10). After calculating the stress values and squared correlations (*R*^2^) for up to four dimensions, we chose a 2-D space (Bieber and Smith, 1986). For the 2-D solutions, the *R*^2^ values for epochs 1 to epoch 4 were 0.868, 0.954, 0.944, and 0.882, respectively. In epoch 1 (Figure 9A), 1 stimulus cluster without the face like-patterns (J1–4) was recognized. In this large cluster, the four stimulus categories (facial photos, cartoon faces, eye-like patterns, and simple geometric patterns) were intermingled. The face-like patterns were located in separate remote places. These data also suggested that, in the first 25-ms period, sSC neurons specifically processed visual information of face-like patterns. In epoch 2 (Figure 9B), four clusters were recognized in which three clusters corresponding to three stimulus categories (i.e., face-like patterns, cartoon faces, and eye-like patterns) were isolated while the simple geometric patterns were distributed around the big cluster of the facial photos. In epochs 3 and 4 (Figures 10A,B), four clusters were recognized, and this was similar to that found in epoch 2. However, in epoch 4, the facial stimuli were grouped into the following two subgroups: frontal faces and profiles. These results indicated that, in the later epochs, the sSC neurons processed more detailed information of the visual stimuli.

### Temporal changes in differential responses and information amounts conveyed by sSC neurons

**Figure 11:**
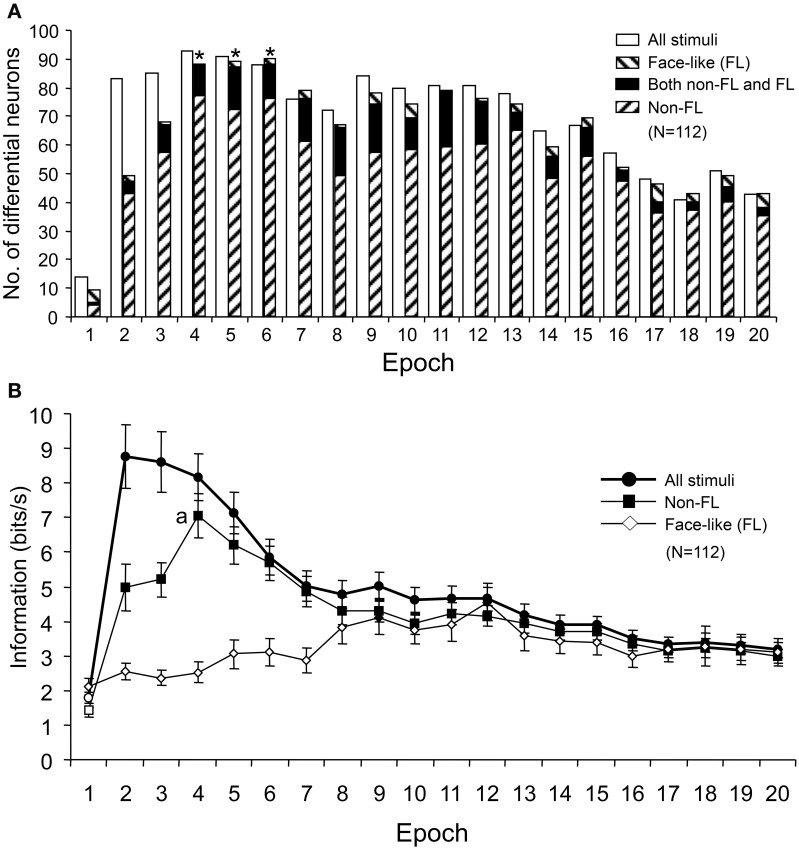
**Changes in the number of differential neurons (A) and the amount of information conveyed by the sSC neurons (B) across the 20 epochs. (A)** The number of differential neurons responding to all visual stimuli and responding to stimuli other than the face-like pattern (Non-FL) increased significantly from epoch 2 compared to epoch 1. ^*^, significant difference from epoch 2 (Bonferroni corrected Fisher's exact probability test, *p* < 0.05). **(B)** Amount of information conveyed by sSC neurons in each epoch. Filled symbols, significant difference from epoch 1 (Bonferroni tests after repeated measures One-Way ANOVA, *p* < 0.05); a, significant difference from epoch 2 (Bonferroni tests after repeated measures One-Way ANOVA, *p* < 0.05).

Figure 11A shows the number of differential neurons (One-Way ANOVA, *p* < 0.05) in each epoch. The numbers of differential neurons were counted for responses to all of the visual stimuli, responses to the face-like patterns (FL), and responses to all visual stimuli except for the FL (Non-FL). The ratios of the differential neurons for responses to all of the visual stimuli and to the Non-FL stimuli were significantly higher in epoch 2 and in later epochs than in epoch 1 (Bonferroni corrected Fisher's exact probability test, *p* < 0.001). Furthermore, the ratios of the differential neurons for responses to the Non-FL stimuli were significantly higher in epochs 4–6 than in epoch 2 (Bonferroni corrected Fisher's exact probability test, *p* < 0.05). However, the ratios of the differential neurons for responses to face-like patterns did not change across the epochs (Fisher's exact probability test, *p* > 0.05). These results indicated that sSC neurons discriminated the Non-FL stimuli more efficiently in the later epochs (epochs 2–4) compared to epoch 1.

The information amounts conveyed by the 112 sSC neurons were separately computed for responses to all of the visual stimuli, responses to the FL patterns, and responses to the Non-FL stimuli. Consistent with the changes in the MDS results and the numbers of differential neurons across the epochs, the amounts of information conveyed by the 112 sSC neurons responding to all of the visual stimuli and responding to the Non-FL stimuli were significantly higher in epoch 2 and in later epochs than in epoch 1 (Bonferroni tests after repeated measures One-Way ANOVA, *p* < 0.05; Figure 11B). Furthermore, in terms of responses to the Non-FL stimuli, the information conveyed in epoch 4 was significantly higher than that in epoch 2 (Figure 11B) (Bonferroni tests after repeated measures One-Way ANOVA, *p* < 0.05). These results indicated that sSC neurons sent more detailed information of the visual stimuli to upstream visual areas in the later epochs.

### Locations of the SC neurons

**Figure 12:**
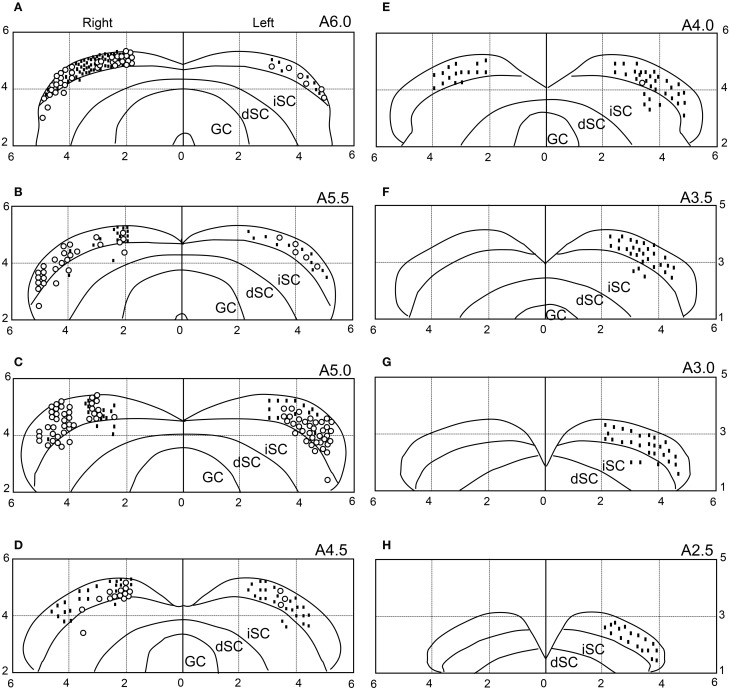
**Recording sites of the 646 SC neurons.** The number in the upper right corner of each section indicates the distance (mm) from the interaural line (anterior posterior level). Horizontal and vertical axes indicate the distance (mm) from the midline and the interaural line, respectively. Open circles, visually responsive neurons; dots, non-responsive neurons. Because some symbols are superimposed, the total number of symbols is smaller than the total number of SC neurons (*n* = 646). iSC, intermediate layers of SC; dSC, deep layers of SC; GC, substantia grisea centralis. **(A–H)** Eight sections from the anterior to posterior SC.

We recorded neuronal activity mainly from the anterior part of the sSC. The histological data indicated that most visually responsive neurons were located in the sSC, while 16 neurons were located in the intermediate layers of the SC. The distributions of the visually responsive (open circles) and non-responsive (dots) neurons are illustrated in Figure 12. Most of the visually responsive neurons were distributed in the anterior area of the sSC.

### Comparison between sSC and pulvinar neuronal responses

**Figure 13:**
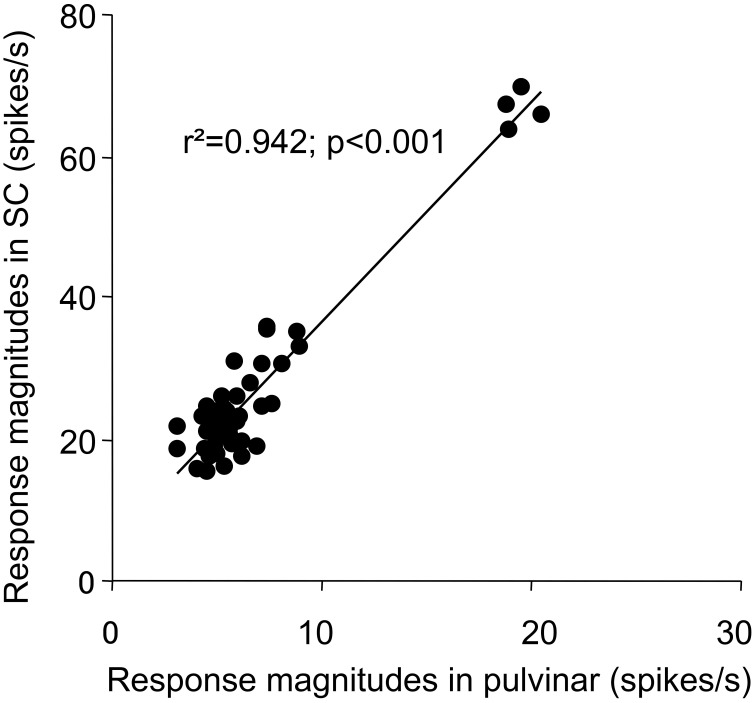
**Correlation plots between response magnitudes to the 49 stimuli in the second (25 to 50 ms) epoch in sSC and those in the first epoch (0 to 50 ms) in the pulvinar**.

Since the SC projects to the pulvinar in the subcortical visual pathway (see Introduction), there might be functional relations between sSC and pulvinar. Therefore, we analyzed correlation between the sSC neuronal responses (present study) and those in the pulvinar (Nguyen et al., 2013), in which the same visual stimuli were presented in the same task. First, the mean response magnitudes to the five stimulus categories in sSC (Figure 5C) tended to be correlated to those in the pulvinar (Pearson's correlation coefficient; *r*^2^ = 0.873, *p* = 0.053). Second, the mean response latencies to the five stimulus categories in sSC (Figure 7B) were significantly correlated to those in the pulvinar (Pearson's correlation coefficient; *r*^2^ = 0.963, *p* < 0.01). Third, the mean response magnitudes to the 49 stimuli in the first (0–25 ms) epoch in sSC (Figure 8A) were significantly correlated with those in the first epoch (0–50 ms) in the pulvinar (Pearson's correlation coefficient; *r*^2^ = 0.862, *p* < 0.001). Fourth, the mean response magnitudes to the 49 stimuli in the second (25–50 ms) epoch in sSC (Figure 13) were also significantly correlated with those in the first epoch (0–50 ms) in the pulvinar (Pearson's correlation coefficient; *r*^2^ = 0.942, *p* < 0.001). Fifth, distance between the stimuli in the MDS space in the first epoch in sSC (Figure 9A) was significantly correlated in that in the MDS space in the first epoch in the pulvinar (Pearson's correlation coefficient; *r*^2^ = 0.850, *p* < 0.001). These results indicated that neuronal response magnitudes and latencies to the visual stimuli in sSC were significantly correlated with those in the pulvinar, and also that both sSC and pulvinar similarly represented the visual stimuli in the MDS space.

## Discussion

### General characteristics of sSC neurons

Visually responsive neurons were located mainly in the anterior part of sSC. Consistently, this region represents a parafoveal region of the visual field (Goldberg and Wurtz, 1972; Boehnke and Munoz, 2008) when visual stimuli were presented on the screen.

The sSC neurons displayed a broad distribution of response latencies. The mean latency of the short-latency group was 52.95 ± 1.71 ms, which was comparable to the findings of previous studies in the SC (Goldberg and Wurtz, 1972; Boehnke and Munoz, 2008; Li and Basso, 2008; White et al., 2009). Because the mean latency in V1, which projects to sSC, is 66 ± 10.7 ms (Schmolesky et al., 1998), some SC neurons with short latencies, especially those with latencies less than 50 ms, might receive inputs directly from the retina.

Our recent study indicated that pulvinar neurons showed visual responses that were comparable to those in the present study; face-like patterns elicited responses with the shortest latencies (Nguyen et al., 2013). Furthermore, the mean response latencies to the visual stimuli were shorter in sSC (present results) than in the pulvinar (65–80 ms) (Benevento and Port, 1995). These results suggested that sSC is one of the important sources of visual inputs to the pulvinar.

### sSC responses to forms

The present results suggest that sSC neurons seemed to differentially respond to visual stimuli although it has been generally accepted that sSC neurons are not sensitive to shape (Schiller and Koerner, 1971; Goldberg and Wurtz, 1972). The latency data may support sSC dependence on stimulus forms in a population level: responses to face-like patterns had the shortest latencies, and scrambling increased response latencies to these stimuli. Consistently, Type II neurons in sSC have relatively large receptive fields, which include the parafoveal area, respond differentially, and strongly to complex forms but poorly to conventional stimuli (stationary or moving white and dark spots and slits) (Rizzolatti et al., 1980), and are located in the deeper layers of sSC (Updyke, 1974). In addition, another study also reported that some SC neurons responded specifically to faces and foods (Arendes, 1994). A non-invasive human study also reported that attention to shape (object) increased the activity of SC (Corbetta et al., 1991). However, response tuning of the individual sSC neurons was very broad in the present study. Even sSC neuronal population activity could not discriminate simple geometrical figures from the facial photos. These results indicated that the discriminability of sSC neurons was limited. Furthermore, although the form-dependent responses were not ascribed to differences in the total luminance and stimulus size in a population level (see Results), this statement is not conclusive in a single neuronal level since complex interaction between a stimulus and a receptive field could affect neuronal responses to a given stimulus in a single neuronal level. Further studies are required to investigate such a complex interaction in sSC.

Unexpectedly, scrambling increased neuronal responses to the stimuli in sSC. Previous studies have reported that the receptive fields for the foveal regions of some sSC neurons were small (Sparks, 1986) and showed center-surround characteristics (Schiller and Koerner, 1971; Updyke, 1974). V1 neurons are sensitive to the higher-order structures of natural scenes, such as image contours due to complex interactions between classic receptive fields and surrounds (Guo et al., 2005). Similar increases in visual responses by scrambling have been reported in the striate cortex (Murray et al., 2002), although response reduction was more common in higher-order areas, such as the prefrontal, superior temporal, and inferotemporal cortices (Bruce et al., 1981; Desimone et al., 1984; Ó Scalaidhe et al., 1999). It has been suggested that perceptual grouping processes take place in the higher-order areas, which send feedback signals to the lower-order areas (V1) in order to reduce responses to incoming sensory signals (Murray et al., 2002, 2004). Therefore, scrambling impedes perceptual grouping, which decreases the feedback signals. These previous findings suggest that similar mechanisms might contribute to the differential responses of the sSC neurons; because sSC receives cortical afferents (see above), the same mechanisms as in V1 might take place in sSC, and a decrease in feedback signals from the higher-order areas might disinhibit sSC. These results suggest that sSC responses to stimulus forms at least in long latencies might be dependent on the cortical feedback signals. This idea is consistent with the present results in which scrambling increased the neuronal responses in long (epoch 3 and later epochs), but not short (epoch 1–2), latencies (Figure 7D). However, this phenomenon (the increase in the firing rates in long latencies by scrambling) is also consistent with the idea that the sSC neurons responded to the part of the images rather than the gestalt structure of the images since the scrambled images contained high-spatial frequency components. Taken together, sSC neurons might be sensitive to both coherency and local feature of the images, which might contribute to visual responses in the upstream areas.

### Role of sSC in the subcortical visual pathway

The SC has been implicated in the innate recognition of biologically relevant objects, such as faces, snakes, prey, and predators (Sewards and Sewards, 2002; Maior et al., 2011, 2012). Consistently, the present results indicated that population activity of the sSC neurons detected face-like patterns with the shortest latencies (epoch 1). Recent studies have suggested that the subcortical visual pathway (SC-pulvinar-amygdala) might convey fast and coarse information of such objects (Johnson, 2005; Day-Brown et al., 2010; Tamietto and de Gelder, 2010; Nakano et al., 2013). Anatomically, the lateral pulvinar receives direct afferents from sSC (Kaas and Lyon, 2007), while the medial pulvinar receives afferents from the deep layers of SC (Benevento and Fallon, 1975; Linke et al., 1999; Grieve et al., 2000), which in turn receives inputs from sSC (Isa, 2002; Doubell et al., 2003). Because the response latencies were as short as 30 ms in the present study, this fast activation of sSC neurons might be attributed to feedforward processing of direct retinal inputs. This information might be conveyed to cortical areas through the pulvinar to induce bottom-up (stimulus-driven) attention to enhance cortical visual processing (Serences and Yantis, 2006). Consistently, the present results indicated that visual responses in sSC were very similar to those in the pulvinar in the population data; neuronal response magnitudes and latencies to the visual stimuli in sSC were significantly correlated with those in the pulvinar, and both sSC and pulvinar similarly represented the visual stimuli in the MDS space especially in short latencies. These findings suggest that, although the present data in sSC could include responses to a part of the images (see above Discussion), all of these responses contribute to visual processing in the upstream areas. Recent neuropsychological studies suggest that this subcortical visual pathway is functional. A blightsight patient with striate cortex damage showed specific activations in the SC, pulvinar, and amygdala in response to angry emotional actions in the blind hemifield (Van den Stock et al., 2011). A patient with bilateral destruction of the visual cortex also showed amygdala activation in response to direct gaze (Burra et al., 2013).

Furthermore, the present study showed that LSF filtering of the images did not affect sSC neuronal responses to face-like patterns and facial photos. This suggested that sSC neurons process LSF information. LSF information is important for face recognition in newborn babies with relatively immature visual cortical areas (Johnson, 2005; de Heering et al., 2008). The face-like patterns used in the present study were the same as those used in the experiment with newborn babies in which the newborn babies preferentially oriented toward such stimuli (Johnson et al., 1991). These face-like patterns were equivalent to LSF components of faces (Johnson et al., 1991). Consistently, LSF components of faces specifically activate the subcortical visual pathway, including SC, pulvinar, and amygdala (Vuilleumier et al., 2003). Furthermore, residual visual ability was tuned to LSF in a patient with blindsight due to lesions in the visual cortical areas (Sahraie et al., 2002).

On the other hand, population activity of the sSC neurons categorized the visual stimuli into 3–5 specific groups in late epochs (especially, epoch 2). Furthermore, the amount of stimulus information conveyed by sSC neurons and the number of stimulus-differential neurons increased in epoch 2. These results indicated that sSC neurons become differentially sensitive to other categories of stimuli in epoch 2 or later, during which cortical neurons also become active [for response latencies of cortical neurons, see a review by Lamme and Roelfsema (2000)]. These findings suggested that differential sSC neuronal population activity in response to various categories of stimuli in epoch 2 might be due to feedback connections from cortical areas.

## Conclusions

Neuropsychological studies have suggested that faces are a special class of objects with particular biological and social significance (Grüsser and Landis, 1991; Carey, 1992) and that facial information is automatically categorized by innate modules in early stages (Vuilleumier, 2000). Our findings suggest that sSC might be a part of this innate module, which has at least the following two functions: providing the circuitry to detect face-like patterns and crude categorization based on connections with cortical areas. Furthermore, sensitivity to LSF visual inputs is consistent to its likely involvement in newborn babies' face recognition skills. The SC therefore may play an important role in ontogenetic development of social cognition.

### Conflict of interest statement

The authors declare that the research was conducted in the absence of any commercial or financial relationships that could be construed as a potential conflict of interest.
